# Mitochondrial AKAP1 supports mTOR pathway and tumor growth

**DOI:** 10.1038/cddis.2017.241

**Published:** 2017-06-01

**Authors:** Laura Rinaldi, Maria Sepe, Rossella Delle Donne, Kristel Conte, Antonietta Arcella, Domenica Borzacchiello, Stefano Amente, Fernanda De Vita, Monia Porpora, Corrado Garbi, Maria A Oliva, Claudio Procaccini, Deriggio Faicchia, Giuseppe Matarese, Federica Zito Marino, Gaetano Rocco, Sara Pignatiello, Renato Franco, Luigi Insabato, Barbara Majello, Antonio Feliciello

**Affiliations:** 1 Department of Molecular Medicine and Medical Biotechnologies, IEOS-CNR, University Federico II, Naples, Italy; 2 I.R.C.C.S Neuromed, Pozzilli, Italy; 3 I.R.G.S., Biogem, Ariano Irpino, Avellino, Italy; 4 Department of Translational Medical Sciences, University Federico II, Naples, Italy; 5 Pathology Unit, Naples, National Cancer Institute, Fondazione Pascale, Naples, Italy; 6 Division of Thoracic Surgery, National Cancer Institute, Fondazione Pascale, Naples, Italy; 7 Department of Advanced Biomedical Sciences, University Federico II, Naples, Italy; 8 Department of Biology, University Federico II, Naples, Italy; 9 Department of Mental and Physical Health and Preventive Medicine, Second University of Naples, Naples, Italy

## Abstract

Mitochondria are the powerhouses of energy production and the sites where metabolic pathway and survival signals integrate and focus, promoting adaptive responses to hormone stimulation and nutrient availability. Increasing evidence suggests that mitochondrial bioenergetics, metabolism and signaling are linked to tumorigenesis. AKAP1 scaffolding protein integrates cAMP and src signaling on mitochondria, regulating organelle biogenesis, oxidative metabolism and cell survival. Here, we provide evidence that AKAP1 is a transcriptional target of Myc and supports the growth of cancer cells. We identify Sestrin2, a leucine sensor and inhibitor of the mammalian target of rapamycin (mTOR), as a novel component of the complex assembled by AKAP1 on mitochondria. Downregulation of AKAP1 impaired mTOR pathway and inhibited glioblastoma growth. Both effects were reversed by concomitant depletion of AKAP1 and sestrin2. High levels of AKAP1 were found in a wide variety of high-grade cancer tissues. In lung cancer, AKAP1 expression correlates with high levels of Myc, mTOR phosphorylation and reduced patient survival. Collectively, these data disclose a previously unrecognized role of AKAP1 in mTOR pathway regulation and cancer growth. AKAP1/mTOR signal integration on mitochondria may provide a new target for cancer therapy.

The cellular energy producing systems reside in mitochondria. Damage to mitochondria leads to ageing and degenerative diseases.^1^ In normal cells, dynamic adjustment of mitochondrial activities promotes metabolic adaptation to changes in extracellular microenvironment and nutrient availability.^2^ In tumor cells, this regulatory system efficiently couples energy production and synthesis of intermediates to enhanced metabolic demands of actively proliferating cells.^3^ Interfering with this regulatory circuit significantly disturbs the growth and progression of human cancer.^4^

Signaling events generated at the cell membrane by hormones and growth factors modulate mitochondrial activity, helping to adapt the cell to changes in metabolic demands. cAMP-dependent protein kinase (PKA) mediates hormone effects on cellular respiration. Localization of PKA at membranes, cytoskeleton and cellular organelles is achieved by direct interaction with A-kinase-anchor-proteins (AKAPs).^5^ AKAPs act as local signal transduction units that direct and amplify cAMP signals at target sites.^6, 7^ AKAP1 targets PKA to the outer mitochondrial membrane.^8^ AKAP121/149, AKAP100 and AKAP84 are alternate splice products of a single gene (AKAP1).^9^ These splice variants share a similar NH_2_-terminal core, which includes the mitochondrial-targeting domain and the PKA-binding domain, but diverge significantly at the C-terminus. AKAP1 binds not only PKA, but also PDE4A,^10^ ser/thr phosphatase (PP1),^11^ transcription factors^12, 13^ and an src-associated tyrosine phosphatase (PTPD1).^14^ The macromolecular complex assembled by AKAP1 efficiently integrates different signaling events, impacting on oxidative phosphorylation, metabolism and survival.^15, 16^ Under hypoxia, proteolysis of AKAP1 promotes mitochondrial fragmentation and a drop in oxidative metabolism, rapidly adapting the ischemic tissue to low oxygen and metabolite availability.^17, 18^ Mouse AKAP121 and the human ortholog AKAP149, have an RNA-binding KH domain motif.^9^ The KH domain binds to nuclear-encoded mRNAs for mitochondrial proteins, constituting signal crossroads for translation and import of proteins into these organelles.^19^ Recent evidence further extends these observations. MDI, the Drosophila ortholog of AKAP1, acts as a major regulator of protein translation. During oogenesis, MDI recruits the translation stimulator La-related protein (Larp) on the mitochondrial outer membrane. MDI-LARP complex promotes the synthesis of a subset of nuclear-encoded mitochondrial proteins required for mtDNA replication and mitochondrial biogenesis.^20^ However, the relevance of AKAP1 in signaling pathways controlling protein translation, as mTOR, and cancer cell proliferation has not so far been elucidated.

mTOR is a member of phosphatidylinositol 3-kinase-related kinase protein family that regulates protein synthesis, transcription, autophagy, growth, motility and survival.^21^ mTOR integrates inputs from pathways activated by insulin, growth factors and amino acids.^22, 23^ It forms two structurally distinct multimeric complexes: mTORC1 and mTORC2. These complexes are targeted to different intracellular compartments and play different roles in cell physiology.^24^ mTORC1 is the principal sensor of nutrients, redox state and energy availability, and controls the anabolic pathway. The presence of amino acids in the cellular microenvironment is necessary for mTORC1 activity and it requires nucleotide loading of the Rag GTPases. The nucleotide loading state of the Rag proteins is controlled by the GAP activity of GATOR1/2 proteins.^25^ GATOR2 enhancement of mTORC1 is inhibited by its interaction with sestrin2, an evolutionarily-conserved stress-inducible protein that suppresses oxidative stress.^26^ The binding of leucine to sestrin2 disrupts sestrin2–GATOR2 interaction and activates mTORC1. Thus, sestrin2 works at the boundary between oxidative stress and the anabolic pathway.^27^

Here, we report that mitochondrial AKAP1 is a novel transcriptional target of Myc which controls the mTOR pathway and cancer cell growth. Upregulation of AKAP1 in high-grade human tumors correlates with enhanced mTOR activation and reduced patient survival.

## Results

### AKAP1 is a transcriptional target of Myc

**Figure 1:**
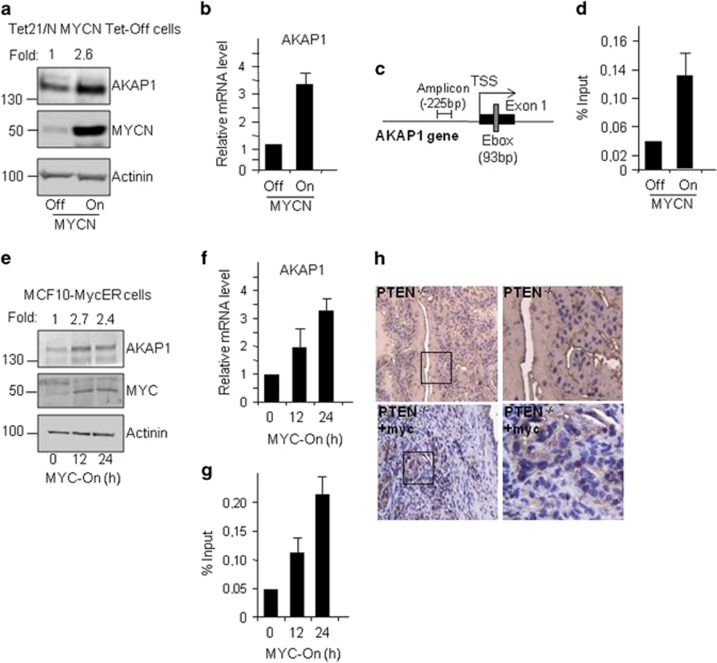
Transcriptional regulation of AKAP1 by Myc. (**a**) Relative levels of AKAP1 and MYCN proteins in Tet21/N MYCN Tet-Off cells with Off (tetracyclin-treated cells) and On (untreated cells) MYCN. Actin was used as a loading control. AKAP1 bands are quantified, normalized against actinin level, and indicated as fold-change (FC) with respect to untreated cells (Off). (**b**) Relative mRNA level of AKAP1 quantified by qRT-PCR analysis in Tet21/N MYCN Tet-Off cells with Off and On MYCN. *P<0,01. (**c**) Simplified structure of the 5’ end of the AKAP1 gene is shown. The amplicon used for ChIP analysis, surrounding the TSS and the Myc-binding site (Ebox), is indicated. (**d**) Chromatin immunoprecipitation assay was performed using MYCN antibody in Tet21/N MYCN Tet-Off cells with Off and On MYCN. Immunoprecipitated samples were analyzed by qPCR using the amplicon indicated in C for the AKAP1 regulatory gene region. Data from three independent ChIP assays were used to determine % of input presented along with S.D., *n*=3. Changes in % input are shown normalized over IgG controls. *P<0,05. (**e**) Relative levels of AKAP1 and MYC proteins were determined by immunoblot in starved cell (0) and at 12 and 24 h after serum+4-OHT treatment in MCF10-MycER cells. Actinin was used for loading control. FC is determined as indicated in a. (**f**) Relative mRNA levels of AKAP1 quantified by qRT-PCR at 0, 12 and 24 h after serum+4-OHT treatment in MCF10-MycER cells. *P<0,01. (**g**) Chromatin immunoprecipitation assay was performed using Myc antibody in MCF10-MycER cells treated for 0, 12 and 24 h with serum+4-OHT. Immunoprecipitated samples were analyzed as indicated in (**d**). *P<0,05. (**h**) Immunohistochemistry analysis for AKAP1 in prostate tissues isolated from transgenic mice carrying a PTEN^−/−^ deletion and overexpressing Myc proto-oncogene under the control of the prostate-specific probasin promoter (PTEN^−/−^ +Myc). Tissue sections of prostate from PTEN^−/−^ mice were used as controls

UCSC genome browser inspection of available ENCODE Data on AKAP1 gene identified a putative Myc-binding domain within the AKAP1 promoter (Supplementary Fig. S1). This finding was in agreement with data collected from gene array analysis indicating the presence of Myc-binding activity at the AKAP1 promoter. Accordingly, we investigated the role of Myc in AKAP1 transcription. First, we determined if MYCN regulates AKAP1 expression in neuroblastoma cells, where MYCN is critical to oncogenesis. To this end, we monitored the relative levels of AKAP1 protein and RNA in the human Tet-21/N neuroblastoma cell line conditionally expressing MYCN under the control of a Tet-Off (tetracycline) promoter. Induction of MYCN (MYCN-On) increased AKAP1 protein (Figure 1a) and AKAP1 mRNA (Figure 1b) by about 2.6-fold and 3.5-fold over the control values (MYCN-Off), respectively.

The data above suggested that MYCN activates AKAP1 expression by direct binding to its promoter. To test this hypothesis, we assessed the relative binding of MYCN to the AKAP1 promoter in MYCN-Off and MYCN-On cells by performing a chromatin immune-precipitation (qChIP) assay.^28^ Chromatin isolated from MYCN-Off and MYCN-On cells was immunoprecipitated with anti-MYCN antibodies and the precipitates were subjected to qPCR using primers surrounding the AKAP1 transcription start site (TSS) (Figure 1c). As shown in Figure 1d, MYCN was detected at the AKAP1 proximal promoter. The relative MYCN occupancy was significantly increased in MYCN-On, compared to MYCN-Off cells, confirming that MYCN directly binds to the AKAP1 promoter.

c-Myc and MYCN proteins share extensive structural and functional similarities.^29^ Hence, we asked if c-Myc also regulated AKAP1 expression. Accordingly, we used a non-transformed mammary epithelial cell line that stably express MycER chimera (MCF10A-MycER). MCF10A-MycER cells, were starved (0) and treated with serum +4-OHT (4-hydrotamoxifen) for 12 and 24 h for c-Myc activation. As shown in Figure 1e, following serum +4-OHT treatment AKAP1 protein (Figure 1e) and mRNA levels increased (Figure 1f). In analogy with MYCN, we determined the c-Myc binding on AKAP1 gene by qChIP experiments. As shown in Figure 1g, we detected c-Myc binding at the AKAP1 proximal promoter.

To further corroborate the regulatory role of Myc in AKAP1 transcription *in vivo*, we monitored the levels of AKAP1 in a mouse model of prostate neoplasia. In this system, transgenic expression of Myc in the prostate of PTEN knockout (PTEN^−/−^) mice driven by the probasin promoter induces neoplastic lesions that recapitulate, in part, those occurring in humans.^30, 31^ As expected, strong AKAP1 staining was evident in the neoplastic lesions compared to the low background staining of the surrounding normal tissue (Figure 1h).

### AKAP1 is overexpressed in human cancers

**Figure 2:**
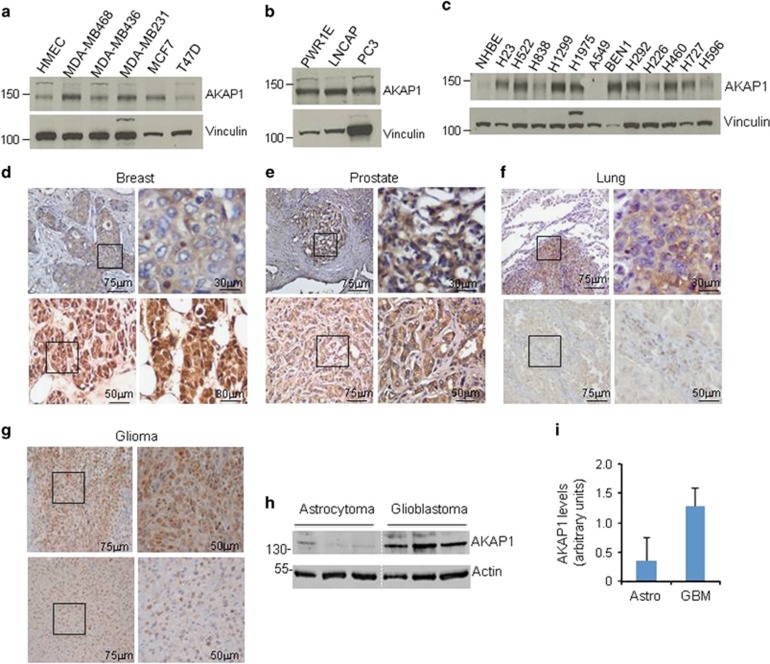
Expression analysis of AKAP1 in human cancer cells and tissues. (**a**–**c**) Immunoblot analysis of AKAP1 in human breast (**a**), prostate (**b**) and lung (**c**) cancer cells. Vinculin was used as a loading control. (**d**–**f**) Immunohistochemistry for AKAP1 in breast (**d**), prostate (**e**) and lung (**f**) high-grade (upper panels, IHC score: 2/3) and low-grade (lower panels, IHC score: 0/1) cancer tissues. Size bars are indicated in the panels. (**g**) IHC on glioblastoma (upper panels, IHC score 3) and astrocytoma (lower panels, IHC score 1) tissues. (**h**) Immunoblot analysis of AKAP1 in lysates prepared from astrocytoma and glioblastoma human tissues. Actin was used as loading control. (**i**) Quantitative analysis of the data shown in (**h**). The data are expressed as mean±S.E.M. of three experiments

The data above indicate that AKAP1 is a transcriptional target of the Myc proto-oncogene. Since Myc is often up-regulated in human tumors, we suspected that AKAP1 might also be highly expressed in cancer. We therefore monitored the levels of AKAP1 in different human cancer cells and tissue samples. Figure 2 shows that AKAP1 is expressed at high levels in a variety of human epithelial cancer cells derived from breast, prostate and lung cancer tissues (Figures 2a–c), compared to non-tumoral cell lines (HMEC and NHBE). Immunostaining analysis revealed high levels of AKAP1 in tissue sections from breast, prostate and lung carcinomas. The staining signal was less pronounced in low-grade tumors (Figures 2d–f and Table 1). We also found marked immunostaining of AKAP1 in high-grade glioma (glioblastoma, GBM), compared to low-grade lesions (astrocytoma) (Figure 2g). Overexpression of AKAP1 in GBM samples was confirmed by immunoblot assay (Figure 2h). Altogether, these findings indicate that AKAP1 represents a novel marker for most of rapidly growing and highly tumorigenic cells.

### AKAP1 regulates mTORC1 pathway

**Figure 3:**
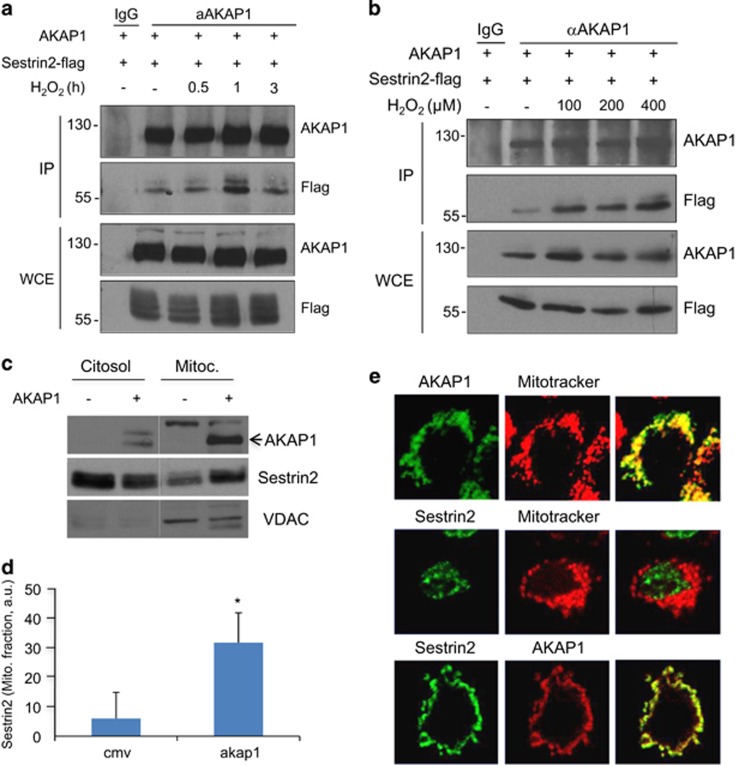
AKAP1 interacts with sestrin2. (**a**) AKAP121 and sestrin2-flag were transiently transfected in HEK293 cells. Cells were treated with H_2_O_2_ (400 *μ*M) for 0.5, 1 and 3  h before collecting. Lysates were subjected to immunoprecipitation with anti-AKAP121 antibody. The precipitates and an aliquot of whole-cell lysates (WCE) were immunoblotted with the indicated antibodies. A representative set of three experiments is shown. (**b**) Same as in **a**, with the exception that the cells were treated for 1 h with the indicated concentrations of H_2_O_2_. (**c**) Mitochondria and cytosolic fractions from HEK293 cells transiently transfected with AKAP1 were immunoblotted with the indicated antibodies. A quantitative analysis is shown. (**d**) The data represent a mean value (±S.E.M.) of three independent experiments (*P<0.05). (**e**) AKAP1-silenced HEK293 (siAKAP1) cells were transiently transfected with AKAP1-V5. Transfected cells were subjected to double immunostaining with anti-sestrin2 and anti-V5 antibodies. Where indicated, cells were labeled with Mitotraker before fixation and visualized by confocal microscopy. A merge composite of the signals is shown (right panels)

A yeast two-hybrid screen using AKAP1 as bait yielded one clone encoding for sestrin2, a stress-induced and antioxidant gene product. Sestrin2 is a negative regulator of the mTOR pathway.^27^ Co-immunoprecipitation assays confirmed that sestrin2 and AKAP1 form a stable complex in lysates (Figure 3a). Given the role of sestrin2 in scavenging the oxidative stress, we asked if sestrin2/AKAP1 interaction is regulated by stress conditions. Cells transiently expressing sestrin2 and AKAP1 were treated for different times and with increasing doses of hydrogen peroxide and subjected to co-immunoprecipitation assays. The induction of oxidative stress by hydrogen peroxide was monitored by analyzing ERK phosphorylation (Supplementary Fig. S2). As shown in Figures 3a and b, the interaction between AKAP1 and sestrin2 was only slightly increased by hydrogen peroxide treatment. Next, we tested if AKAP1 targets sestrin2 to mitochondria by copurification assays and immunostaining analysis. Indeed, Figure 3c shows that expression of AKAP1 increased the amount of endogenous sestrin2 that co-purified with mitochondria (Figures 3c and d). Moreover, double immunostaining analysis confirmed that AKAP1 was required, at least in part, for sestrin2 localization on mitochondria (Figure 3e).

**Figure 4:**
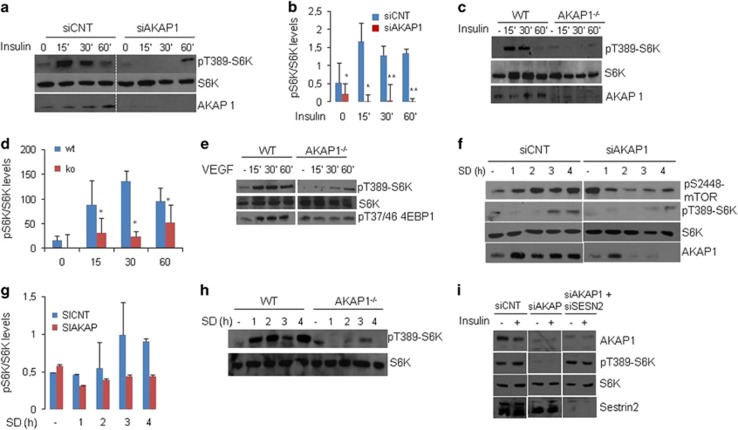
AKAP1 is required for mTOR pathway activation. (**a**) U87MG cells transiently transfected with siRNAs targeting AKAP1 were serum-deprived overnight and then stimulated with insulin for the indicated time points. Lysates were immunoblotted with anti-phosphoT389-pS6K, anti-S6K and anti-AKAP1 antibodies. (**b**) Quantitative analysis of the experiments shown in (**a**). Data represent a mean±S.E.M of four experiments. A mean value±S.E.M of four independent experiments is shown. *P<0.05 *versus* control siRNA. **P<0.005 *versus* control siRNA. (**c**–**e**) Primary endothelial cells from control and AKAP1^−/−^ mice were serum-deprived overnight and then stimulated with insulin (**c**) or VEGF (**e**) for the indicated time points. Lysates were immunoblotted with the indicated antibodies. (**d**) Quantitative analysis of the experiment shown in (**c**). The data are expressed as mean±S.E.M. of three experiments. *P<0.05 between wildtype (wt) and KO cells. (**f**) Same as in **a**, with the exception that transfected U87MG cells were serum-deprived for the indicated time points. Lysates were immunoblotted for pS2448mTOR, phosphoT389-pS6K, anti-S6K and AKAP1. (**g**) Quantitative analysis of the experiments shown in (**f**). The data are expressed as mean±S.E.M. of three experiments. (**h**) Wt and AKAP1^−/−^ endothelial cells were serum deprived for the indicated times. Lysates were immunoblotted with anti-phosphoT389-pS6K, anti-S6K antibodies. (**i**) U87MG cells transfected with control siRNA or with siRNAs targeting AKAP1 and sestrin2 were serum-deprived overnight and then stimulated with insulin (30 min). Lysates were immunoblotted with the indicated antibodies. A representative set of two experiments is shown

In view of the inhibitory role of sestrin2 in mTORC1 pathway, we asked if AKAP1 modulates mTORC1 activity by interacting with sestrin2. As a readout of mTORC1 activity, we monitored phosphorylation of p70S6K at threonine 389 in response to insulin stimulation.^32^ Figures 4a and b show that insulin markedly increased p70S6K phosphorylation as early as 15 min post-treatment, which was followed by a decline in phosphorylated p70S6K levels at later time points. Importantly, phosphorylation of p70S6K was dramatically impaired in cells devoid of AKAP1 (Figures 4a and b). Since Myc induces expression of AKAP1, we tested if Myc also activates mTORC1. As suspected, overexpression of MYCN enhanced p70S6K phosphorylation (Supplementary Fig. S3).

We replicated the experiments in primary cultures of endothelial cells isolated from an AKAP1 knockout mouse. Figures 4c and d show that AKAP1 is, indeed, required for insulin-induced p70S6K phosphorylation. AKAP1 KO also affected mTORC1 activation in response to VEGF treatment (Figure 4e), as shown by decreased phosphorylation of p70S6K and of 4EBP at threonine 37/46 (Figure 4e). Next, we tested the role of AKAP1 in mediating the activation of mTOR pathway in response to serum deprivation. Figures 4f and g show that in control cells, serum-deprivation-induced phosphorylation of mTOR at its active site (S2448) and of p70S6K at thr 389. Genetic silencing of AKAP1 dramatically decreased phosphorylation of both kinases (Figures 4f and g). We replicated these experiments in primary endothelial cells (Figure 4h). We found marked induction of S6K phosphorylation in serum-deprived cells, starting 1 h from serum starvation and peaking 2–4 h later. Deletion of AKAP1 nearly abolished phosphorylation of p70S6K in serum-deprived cells. To prove that AKAP1 regulates mTOR pathway by interfering with the inhibitory activity of sestrin2, we measured p70S6K phosphorylation in cells subjected to a double-knockdown of AKAP1 and sestrin2. Figure 4i and Supplementary Fig. S4 shows that concomitant downregulation of AKAP1 and sestrin2 restored, at least in part, the insulin-induced p70S6K phosphorylation in AKAP1-silenced cells.

### AKAP1 supports tumor growth

**Figure 5:**
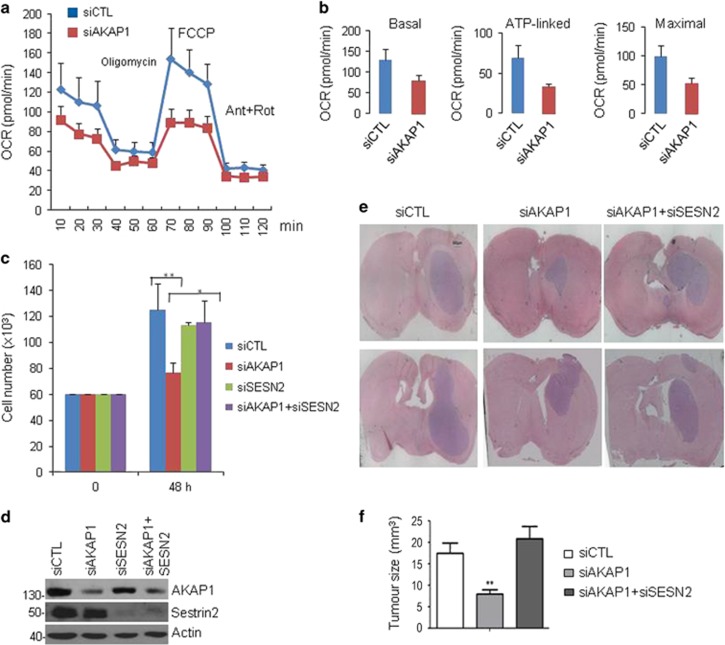
AKAP1 is required for oxidative metabolism and GBM cell growth. (**a**) Kinetic profile of oxygen consumption rate (OCR) in U87MG cells silenced for AKAP1 compared to controls. The data are shown as mean±S.E.M. of three independent experiments, each of them in technical triplicates derived from the same number of seeded cells (2 × 10^4^/well). OCR was measured in real time, under basal conditions, and in response to the indicated mitochondrial inhibitors: oligomycin, FCCP, Antimycin A and Rotenone. (**b**) Indices of mitochondrial respiratory function, calculated from the OCR profile of U87MG cells: basal OCR, ATP-linked OCR and maximal OCR in control and AKAP1-silenced cells. Data are expressed as mean±S.E.M. of three measurements deriving from three independent experiments, each of them in technical triplicates. *P=0,048 (basal), ***P=0,0007 (maximal). (**c**) U87MG cells were transiently transfected with control siRNAs or with siRNAs targeting AKAP1 and sestrin2. Following transfection, cells were seeded in multiwell plates, collected and counted. A mean value±S.E.M. of three experiments is shown. (**d**) Lysates from siRNAs-transfected cells were immunoblotted for AKAP1, sestrin2 and actin. (**e**) U87MG cells transiently transfected with the indicated siRNAs were stereotaxically implanted into the brain (left caudate nucleus) of CD1 nude mice. Tissue coronal sections from tumor lesions were stained with hematoxylin/eosin; scale bar 500 *μ*m. (**f**) Volumetric analysis of tumor brain in nude mice. Number of animals tested: control siRNA: *n*=8; *AKAP1* siRNA: *n*=8; *AKAP1*+sestrin2 *n*=8. **P<0.05 control siRNA *versus AKAP1* siRNA

Activation of the mTOR pathway has been linked to tumor growth and its inhibitors are currently used in clinical trials for cancer patients.^33^ Since AKAP1 is critical for mTORC1 activation, we predicted that the anchor protein supports tumor cell growth. As a model system, we used human glioblastoma multiform cells (U87MG). AKAP1 stimulates mitochondrial respiration,^16^ so we analyzed the effects of AKAP1 silencing on the oxidative pathway in GBM cells. As readout of mitochondrial oxidative capacity, we tested the oxygen consumption rate (OCR) of the cells under basal conditions and in the presence of oligomycin (ATP sintase inhibitor), carbonylcyanide-4-(trifluoromethoxy) phenylhydrazone (FCCP) (a mitochondrial protonophore uncoupler), as well as rotenone and antimycin A (two mitochondrial transport chain inhibitors). This treatment allowed us to estimate the contribution of AKAP1 inhibition on basal and maximal respiration and ATP-linked OCR. Figures 5a and b show that downregulation of endogenous AKAP1 severely affected mitochondrial oxidative capacity, compared to controls. Next, we assessed the contribution of AKAP1 and sestrin2 to GBM growth. Figure 5c shows that downregulation of AKAP1 decreased the growth of GBM cells in culture. The effects of AKAP1 silencing were reversed by concomitant downregulation of sestrin2 (Figure 5c). Next, we assessed the contribution of AKAP1 and sestrin2 in brain tumor development by using an orthotopic glioblastoma mouse model.^34^ In this system, transient siRNA-mediated silencing of target genes is capable of delaying cell’s growth at early days post-implantation, and this is sufficient to impact tumor growth at the end point of the experiment. U87MG cells were transiently transfected with siRNA targeting either AKAP1, sestrin2 and both. A non-targeting siRNA was used as control. The efficiency of silencing was monitored by immunoblot analysis (Figure 5d). Transfected cells were, then, stereotaxically implanted into the left caudate nucleus of mouse brain. Three weeks later, the mice were sacrificed. Histological analysis of post-mortem control mouse brain revealed an homogenous tumor mass with sharp borders that was delimited from the adjacent normal brain tissue and composed of large pleomorphic cells with abundant eosinophilic cytoplasm. (Figure 5e). Downregulation of AKAP1-reduced tumor weight by about 2.5-fold. Concomitant downregulation of sestrin2 restored tumor growth generated from AKAP1-silenced cells. These findings support a role of AKAP1 in buffering the inhibitory effects of sestrin2 on mTORC1 and tumor growth.

### AKAP1 overexpression inversely correlates with patient survival

**Figure 6:**
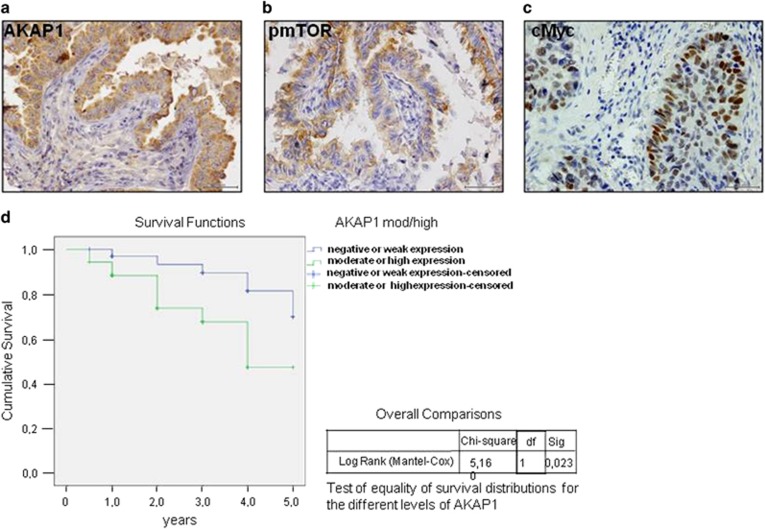
Expression analysis of AKAP1, phospho-mTOR and c-Myc in lung cancer tissues. (**a**) Strong cytoplasmic staining of AKAP1 (40X); (**b**) high cytoplasmic staining of phospho-mTOR (40 × ); (**c**) high nuclear staining of c-Myc (40 × ). (**d**) Kaplan–Meier curve analysis to evaluate overall survival according to AKAP1 expression in 87 NSCLC patients (57 with high/moderate and 30 with weak/negative AKAP1 staining)

The data above indicate that AKAP1 is a transcriptional target of myc and is overexpressed in a wide array of human tumors. Given its positive role in mTOR pathway and tumor growth, we investigated the expression profile of AKAP1 and its correlation with myc and mTOR in human tumor tissues. A validated tissue microarray (TMA) containing tumor samples derived from patients that underwent to surgery for non-small cell lung cancer (NSCLC) was subjected to immunostaining analysis for AKAP1. The details of the clinical and pathological features of 87 NSCLC patients tissues with AKAP1 expression are reported in Supplementary Fig. S5a. The immunohistochemical results showed moderate/high expression of AKAP1 in 57 out of 87 tumors and weak staining in the remaining 30 tissues. Most cases with high/moderate levels of AKAP1 staining were adenocarcinoma (47/87) and the statistical analysis showed a significance association between AKAP1 expression and adenocarcinoma histotype (*P*=0.004). In addition, we performed immunostaining analysis for phospho-mTOR and c-Myc. The results showed that 22 out of 87 NSCLC patients were concomitantly positive for phospho-mTOR and AKAP1 (Figures 6a and b). The statistical analysis showed a trend of association between both staining signals (*P*-value=0.075) (Supplementary Fig. S5b). The results of c-Myc staining revealed that 10 cases expressed both c-Myc and AKAP1, although this falls below a significant correlation (Figure 6c and Supplementary Fig. S5c). Kaplan–Meier curve analysis suggested that AKAP1 expression influences the prognosis of NSCLC patients, as moderate/high expression of AKAP1 was significantly associated with a worst prognosis (*P-*value=0.023) (Figure 6d).

## Discussion

We provide evidence that mitochondrial AKAP1 is a novel Myc transcriptional target and is highly expressed in a wide array of human cancer tissues. AKAP1 is required for tumor cell growth *in vitro* and *in vivo*. At a mechanistic level, we found that AKAP1 interacts with sestrin2, a stress-induced gene product and negative regulator of the mTORC1 pathway. By suppressing the negative loop imposed by sestrin2 on the mTOR pathway, AKAP1 supports mTORC1 activation.

Intracellular targeting of enzymes, adapters, regulators and effectors of signaling pathways have emerged as an important mechanism that tightly control key biological activities. AKAPs are a large family of structurally different, but functionally homologous proteins that regulate signal transduction events generated by G-protein coupled receptors.^35, 36^ Different partners and regulators have been identified in complex with AKAPs and their role in specialized cell functions has been experimentally addressed.^37, 38^ Tissue-specific expression of AKAPs by modulating the distribution of signaling complexes at distinct intracellular compartments further contributes to differentiate the biological responses of distinct tissues to hormones and growth factors.^39^ AKAP1 scaffolds a local transduction unit that operates at the OMM to control different aspects of the organelle physiology. Under hypoxic conditions, ubiquitin-directed proteolysis of AKAP1 attenuates mitochondrial respiration and promotes mitochondria fission. This is of importance in the course of ischemic insult, where it serves as a mechanism to preserve cell viability and tissue remodeling under hypoxic conditions.^17^

Here, we demonstrate that transcription of AKAP1 is regulated by the Myc proto-oncogene. We identified a cis-acting element within the AKAP1 gene promoter that mediates the interaction with- and the transcriptional regulation by- Myc, both *in vitro* and *in vivo*. It is well known that in dividing cells, Myc links the programmed expansion of mitochondrial content, a process known as mitochondria biogenesis, to the cell cycle. This mechanism ensures sufficient energy (ATP) to the anabolic processes that support the synthesis of the building blocks for the dividing cells.^40^ A wide number of nuclear-encoded genes required for mitochondria biogenesis and activity have been identified as Myc targets.^41, 42^ However, if or how Myc regulates the synthesis of AKAP scaffolds involved in cancer-related mitochondrial activities remained largely unexplored. Our findings added a novel layer of complexity of Myc action in the control of mitochondrial activity. Transcriptional regulation of AKAP1 by Myc would provide an energetic advantage to cancer cells, enhancing energetic metabolism and the anabolic pathway. In this context, high levels of AKAP1 were detected in wide array of human cancer cells and tissues, suggesting a role of this protein in the metabolic processes of actively proliferating cells. The data presented here support this hypothesis. Thus, genetic silencing of AKAP1 severely impaired oxidative metabolism and growth of cancer cells. In xenograft orthotopic mouse models of glioblastoma, downregulation of AKAP1 significantly reduced cancer cell proliferation and tumor development.

At mechanistic levels, we identified sestrin2 as a novel interactor of AKAP1. Sestrin2 was originally identified as the principal p53-induced scavenging system of stressed mitochondria.^43^ In the course of oxidative stress, p53 increases the transcription of sestrin2, which in turn regenerates over-oxidized mitochondrial peroxiredoxins, the enzymes that metabolize peroxides. By reducing oxidized peroxiredoxin levels, sestrin2 acts as a major modulator of antioxidant defense in mitochondria.^44^ Sestrin2 also acts as regulator of anabolic pathways. Under stress conditions, sestrin2 interacts with GATOR2, a component and positive regulator of mTORC1 complex. Sestrin2–GATOR2 interaction prevents the assembly of active mTORC1 complex and activation of the downstream anabolic pathway. Sestrin2, therefore, constitutes at link between p53, genotoxic stress and mTOR pathway.^26^ Here, we found that AKAP1 interacts with and targets sestrin2 to the outer mitochondrial membrane. By reducing the cytoplasmic levels of sestrin2, AKAP1 removes the inhibitory constraint of sestrin2 on metabolism, facilitating mTOR activation and cancer cell growth. AKAP1/sestrin2 complex might also contribute to redox control of mitochondrial compartment. Accordingly, downregulation of AKAP1 promotes oxidative stress and mitophagy, eventually leading to apoptosis.^8, 15^ The recent identification of MDI, the Drosophila ortholog of AKAP1, as a master regulator of the protein translation machinery during oogenesis further supports a major role of AKAP1 in the control of anabolic processes.^20^ By regulating oxidative phosphorylation, antioxidant responses and mTOR activity, AKAP1 efficiently couples energy production and scavenging system to protein translation, contributing to growth advantage of rapidly proliferating cells.

Taken together, our findings demonstrate that AKAP1 is a novel Myc transcriptional target that controls mTOR pathway. The complex assembled by AKAP1 on mitochondria is particularly relevant for cancer cells, as it provides sufficient energy for the synthesis of the building blocks of cellular organelles. Interfering with the signaling events regulated by AKAP1 at mitochondria and modulating its interaction with components of the metabolic pathways could provide novel molecular targets for cancer therapy.

## Materials and Methods

### Cells and tissues

The human embryonic kidney cell line (HEK293) was cultured in Dulbecco’s modified Eagle’s Medium (DMEM) containing 10% fetal bovine serum (FBS) in an atmosphere of 5% CO2. The human glioblastoma cell line U87MG (grade-IV) and the human epithelial cancer cells, derived from breast, prostate and lung cancer tissues were purchased from the American Type Culture Collection (ATCC) and maintained in modified Eagle’s medium supplemented with 10% heat inactivated fetal calf serum and 2 mM L-glutamine, 100 IU/ml penicillin, 100 *μ*g/ml streptomycin, 1 × non-essential amino-acid, 1 mM sodium pyruvate, at 37 °C, 5% CO2 and 95% of humidity. MCF10A were cultured in 1:1 mixture DMEM-F12 supplemented with 5% horse serum, 10 *μ*g/ml insulin, 0,5 *μ*g/ml hydrocortisone, 100 ng/ml cholera enterotoxin, and 20 ng/ml epidermal growth factor, and incubated at 37 °C in humidified atmosphere with 5% CO2. MCF10a-MycER cells were starved by growth in minimal medium (1:1 mixture DMEM-F12 supplemented with 5% horse serum) for 2 days. Myc induction was obtained by treating the cell for the indicated time with complete medium with 600 nM of 4-hydroxytamoxifen (OHT). SHEP Tet-21/N MYCN Tet-Off cells were cultured in DMEM supplemented with antibiotics and 10% FBS. MYCN expression under control of the Tet-Off system was turned off by the addition of tetracycline 1 *μ*g/ml for 1 week.

### Antibodies and chemicals

A polyclonal antibody directed against murine AKAP1 was raised as described before.^17^ Anti-Flag antibody was purchased from Sigma (St. Louis, MO, USA), anti-pT389-S6K, anti-pS2448mTOR and anti-sestrin2 were purchased from Cell Signaling (Danver, MA, USA). Anti-S6K antibody was purchased from Santa Cruz Biotechnology (Dallas, TX, USA) and anti-human AKAP1 antibody from Bethyl (Bethyl Montgomery, TX, USA).

### Transfection of plasmids and siRNAs

Vectors encoding for wildtype or mutant AKAP1 were previously described. Sestrin2-flag was kindly provided by Dr. Peter Chumakov and described previously.^45^ Transfection efficiency was monitored by including a GFP vector in the transfection mixture. ON-TARGET plus siRNA targeting coding regions of human AKAP1 and a ON-TARGET smart-pool siRNA targeting sestrin2 were purchased from Dharmacon (Lafayette, CO, USA). The siRNA sequence (Thermo Scientific) targeting human AKAP1 is the following: GGGAGCAUGUCUUGGAAUU. The following are the siRNA sequences (Thermo Scientific, Waltham, MA, USA) targeting human sestrin2: sequence 1: GUUUUGAGCUGGAGAAGUC; Sequence 2: CCAUCAAUGUGAAAGUUGG; Sequence 3: GCAGCCUGUUCUUUGGUUA; Sequence 4: UCUUUGGCAUCAGAUACGA. siRNAs were transiently transfected using Lipofectamine 2000 (Invitrogen, Carlsbad, California, USA) at a final concentration of 100 pmol/ml of culture medium. For siRNA experiments, similar data were obtained using a mixture or four or two independent siRNAs.

### Immunoprecipitation and western blot analysis

Cells were washed twice with phosphate-buffered saline and lysed in Tris-buffered saline buffer-1% Triton-X 100 (NaCl, 150 mM; Tris-HCl, 50 mM, ph 7,5; EDTA, 1 mM; NaF, 1 mM; Na4P2O7, 1 mM; Na3VO4, 0.4 mM). Lysed cells of 1.5 mg was subjected to immunoprecipitation with the indicated antibodies. Whole-cell lysates (100 *μ*g) and immunoprecipitates were resolved on sodium dodecyl sulfate polyacrylamide gel and transferred on nitrocellulose membrane (Biorad, Milan, Italy) for 3 h. Filters were blocked for 1 h at room temperature in Tween-20 Phosphate buffer saline (TPBS) (PBS- Sigma, 0,1% Tween 20, pH 7.4) containing 5% non-fat dry milk. Blots were then incubated O/N with primary antibody. Blots were washed three times with TPBS buffer and then incubated for 1 h with secondary antibody (peroxidase-coupled anti-rabbit (GE-Healthcare, Little Chalfont, UK) in TPBS. Reactive signals were revealed by enhanced ECL western blotting analysis system (Roche, Basilea, Swizerland).

### qRT-PCR

RNA was extracted from MCF10-MycER and Tet-21/N cells using EuroGold Trifast (EuroClone, Milan, Italy). cDNA was generated using Quantitec Reverse Transcription Kit (Qiagen, Hilden, Germany), according to manufacturer’s protocol. Quantitative analysis was performed using SYBR Green 2X PCR Master Mix (Applied Biosystem, Forster City, CA, USA). Each sample was run in triplicate and normalized to the expression of housekeeping beta-glucoronidase (GUS) gene as previously described.^46^ The primers used in qPCR are: AKAP1, TTCTCTGCCGATGACATCCT and CATTGACCTGGTTGACC ACA; GUS GGAATTTTGCCGATTTCATGA and CCGAGTGAAGATCCCCTTTTT.

### Chromatin immunoprecipitation

Chromatin assays were performed as described.^46^ In brief, 1 × 10^6^ cells were cross-linked using formaldehyde to a final concentration of 1% and the reaction was stopped by adding 0.125M Glycine. Cell pellet was resuspended in Cell Lysis Buffer and after 6000 r.p.m. centrifugation RIPA buffer were added to perform nuclei lysis. DNA shearing was conducted by sonication using Bioruptor (Diagenode, Serainge, Belgium). A small aliquot of sonicated material was put aside and remaining sample immunoprecipitated using 5 micrograms of following antibodies: anti-c-Myc (N262) and anti-MYCN (B8.4.B) from Santa Cruz. Rec-sepharose Protein A or G beads (Invitrogen) were used to immobilize immunocomplexes and after RNAse-A treatment (37 °C 1 h) reverse cross-linking were performed using Proteinase K (Roche) for 6 h at 65 °C. Immunoprecipitated DNA was purified using Phenol/Chloroform and Ethanol precipitation techniques. DNA was analyzed by qPCR using the following primers: GGTTGACCCTTCGAGACAAG and CAACCGGAGGAACCTTGAT.

### Implantation of U87MG cells in mouse brain

Male CD1 nude mice (Charles River Laboratories, Wilmington, MA, USA; 20–22 g body weight) were housed in controlled conditions (temperature 22 °C; humidity 40%) on a 12-h light/dark cycle with food and water ad libitum and observed daily. The experiments were performed according to the guidelines for the care and use of animals promulgated by the National Institutes of Health (Bethesda, MD, USA). Forty-eight hours before implantation, U87MG cells were transfected with control siRNA or siRNAs targeting AKAP1 and sestrin2. The cells (4 × 10^5^ cells/animal) were then resuspended in DMEM without FCS implanted and implanted in the caudate nucleus of the mice using the following stereotactic coordinates: 0.6 mm anterior to the bregma, 1.7 mm lateral to the meridian line, 3.5 mm in depth from the surface of the skull. Animals were anesthetized intraperitoneally with ketamine (100 mg/kg) and xylazine (10 mg/kg). Twelve days after inoculation, the mice were sacrificed by cervical dislocation after anesthesia, and their brains removed, fixed in formalin, dehydrated in ethanol at increasing concentrations and embedded in paraffin. From each brain, 10-m-thick serial sections, from the beginning of the striatum to the hippocampus (1 section every 400 *μ*m), were sliced. Subsequently, the sections were stained with Mayer’s hematoxylin and eosin (both from Diapath, Bergamo, Italy) and subjected to analysis for the quantification of tumor volume. The volumetric analysis was performed using software that measures the tumor area in each section and calculates the total volume of the tumor according to the Cavalieri method13 by using the following formula: *V*= S(A)i x TS x n., where (*A*)_i_ is the area of the tumor in level i, *T*_S_ is the section thickness, and *n* is the number of sections disposed between the 2 levels.

### Immunohistochemistry and immunofluorescence analysis

Briefly, formalin-fixed and paraffin-embedded tissue sections (4 *μ*m) were deparaffinized in xylene and rehydrated with graded ethanol. 60 samples of prostatic tumors, 10 cases of benign prostatic hyperplasia and 10 cases of breast carcinomas were collected from the archive of the dept of Advanced Medical Science, section of the Anatomic Pathology, University Federico II Naples-Italy. Relatively to the prostatic tumors 30 cases were high-grade PCa of which 20 cases with 4+4=8 Gleason score, and 10 cases with 5+4=9 Gleason score. Thirty cases were low-grade PCa with 3+3=6 Gleason score. Moreover, 10 cases of benign prostatic hyperplasia were selected. About the breast carcinomas, 5 cases were high-grade invasive ductal carcinomas and 5 cases were low-grade invasive ductal carcinomas. Bioptic samples of GBM were surgically removed from Neuromed patients. All patients gave their informed consents and were shown to carry glioblastoma multiforme (according to WHO classsification). All tumors were positive for vimentin, GFAP (glial fibrillary acidic protein) and EGFR. Immunostaining for AKAP1 of all cases was evaluated. Antigen retrieval was carried out in citrate buffer (pH=6.0, 12 min, microwave oven). Endogenous peroxidase activity was quenched with 0.3% hydrogen peroxide for 12 min. Non-specific-binding sites were blocked with 5% normal horse serum in TBS-Tween (Wash buffer, Dako, Glostrup, Denmark) for 30 min. Sections were incubated with primary antibody (AKAP1) overnight at 4 °C. All sections were visualized using the Liquid DAB Substrate Chromogen System for peroxidase (DakoCytomation) and were counterstained with hematoxylin, dehydrated and mounted. In particular, a score of 0–3 was given: 0, negative staining; 1, weak expression in 20% of the cytoplasm of tumor cells; 2, moderate or strong expression in 20–75% of tumor cells; 3, strong expression in>75% of tumor cells. A score of 0 or 1 was considered as negative, and a score of 2 or 3 was considered positive (Tab). For immunofluorescence studies, U87MG cells transiently transfected with AKAP1 and sestrin2-flag were plated on poly-L-lysine (10 *μ*g/ml) coated glass coverslips. Cells were fixed and immunostained with anti-Flag and anti-AKAP1 antibodies. Immunofluorescence was visualized using a Zeiss LSM 510 Meta argon/krypton laser scanning confocal microscope. Quantification of the immunofluorescent images and correlation (Pearson's) coefficient were calculated by Image-J software (National Institute of Health, USA).

### TMA building

A total of 94 NSCLC selected from 2006 to 2010, at the National Cancer Institute 'Giovanni Pascale' of Naples, were used for building a retrospective prognostic TMA. A prognostic TMA was designed in order to allow high-throughput expression profiling of several tumor samples linked to clinicopathological databases. The TMA was built using two cores from different areas selected on the H&E-stained slides and, whenever possible, one core of normal tissue of the same tissue block. Tissue cylinders with a diameter of 1 mm were punched from morphologically representative tissue areas of each 'donor' tissue block and brought into one recipient paraffin block (3 × 2.5 cm) using a semiautomated tissue arrayer (Galileo TMA). A standard protocol was used for the immunostaining of the paraffin-embedded samples to evaluate the expression of AKAP1, phospho-mTO, and c-Myc. An appropriate external positive control tissue was used for each staining procedure; the negative control consisted of performing the entire IHC procedure on an adjacent section in the absence of the primary antibody. Briefly, paraffin slides (4 *μ*m) were cut, deparaffinized in xylene and rehydrated through graded alcohols. Antigen retrieval was performed with slides heated in EDTA buffer (pH 9.0) or citrate buffer (pH 6) in a bath for 20 min at 97 °C. The endogenous peroxidase was inactivated with 3% hydrogen peroxide and then the protein block (BSA 5% in PBS 1 × ) was performed. Tumor sections were incubated with the primary antibodies according to the specific conditions tested for each one: anti-AKAP1 (#sc-6439, Santa Cruz Biotechnology, dilution 1:100); rabbit polyclonal anti-mTOR phosphoS2448 (Abcam, Cambridge, UK; ab1093 dilution 1:50); rabbit monoclonal anti-c-Myc Y69 (#ab32072, Abcam dilution:1:200). The tissue sections were incubated with polyclonal goat anti-rabbit (#ab150077; Abcam; dilution, 1:400), goat anti-mouse (#ab150115; Abcam; dilution, 1:400) and donkey anti-goat (#ab7125, Abcam; diluition:1:400) IgG biotinylated secondary antibodies for 40 min at room temperature. Immunoreactivity was visualized by means of avidin-biotin-peroxidase complex kit reagents (Novocastra, Newcastle, UK) as the chromogenic substrate. Finally, the sections were developed with diaminobenzidine and counterstained with hematoxylin. Stained slides were analysed by two independent observers using an optical microscope (Olympus BX41). Expression of the biomarkers was evaluated semiquantitatively based on the number of immunoreactive cells and staining intensity. The proportion of staining was scored on a scale from 0 to 3 as follows: diffuse, ≥50% positive (score 3); regional, 10–49% positive (score 2); focal, 1–9% (score 1); and negative (score 0). IHC scoring was based on the cytoplasmic-staining intensity for anti-AKA149 and anti-mTOR phosphoS2448, and nuclear-staining for c-Myc, as follows: no staining, score 0; weak, score 1+ moderate, score 2+ high, score 3+.

### Statistical analysis

Pearson *X*-square test was performed to determine the association of clinical characteristics with status of protein expression. The Spearman rank test was used to assess the correlation between protein phenotypes. *P*<0.05 (2-sided) was considered to be statistically significant. Overall Survival (OS) curves were calculated using the Kaplan–Meier method. OS was defined as the time from histological diagnosis to death by any cause or until the most recent follow-up. The log-rank test was used to compare survival distributions between positive and negative staining groups. Data analysis and summarization were conducted using SPSS 20.0 for Mac (SPSS Inc., Chicago, Il, USA). Statistical analysis of tumor volume in CD1 nude mice was performed using Student’s *t*-test. All data were expressed as mean±S.E.M. Statistical comparisons between experimental groups were performed using the *t*-test or one-way analysis of variance when required. *P*<0.05 was considered statistically significant.

### Metabolic assays

The metabolic profile was monitored in control U87MG cells or in cells subjected to AKAP1 silencing. Real-time measurements of OCR were made using an XF-96 Extracellular Flux Analyzer (Seahorse Bioscience, North Billerica, MA, USA). Cells were plated in XF-96 plates (Seahorse Bioscience) at the concentration of 2 × 10^4^ cells/well and cultured for the last 12 h in DMEM, 10% FBS. OCR was measured in XF media (non-buffered DMEM medium, containing 10 mM glucose, 2 mM L-glutamine, and 1 mM sodium pyruvate), under basal conditions and in response to 5 *μ*M oligoMycin, 1.5 *μ*M of FCCP and 1 *μ*M of AntiMycin and Rotenone (all from Sigma Aldrich). Indices of mitochondrial respiratory function were calculated from OCR profile: basal OCR (before addition of oligomycin), ATP-linked OCR (calculated as the difference between basal OCR rate and oligomycin-induced OCR rate) and maximal OCR (calculated as the difference of FCCP rate and antimycin+rotenone rate).

## Figures and Tables

**Table 1:** Correlation of Gleason score and AKAP1 expression in PCa and BPH

**Cases**	**GS**	**AKAP1 staining**
HG-PCa 20	4+4=8	High
HG-PCa 10	5+4=9	High
LG-PCa 30	3+3=6	Low
BPH 10	NA	Low

Abbreviations: PCa, prostatic carcinoma; HG, high-grade; LG, low-grade; BPH, benign prostatic hyperplasia; NA, not applicable

AKAP1 staining by immunohistochemistry
