# Supplementary material for: Mitochondrial AKAP1 supports mTOR pathway and tumor growth
Source: Cell Death Dis. 2017 Jun 1;8(6):e2842–. doi: 10.1038/cddis.2017.241 (PMC5520900; doi:10.1038/cddis.2017.241)
Supplement: Supplementary Information [file cddis2017241x1.pdf]

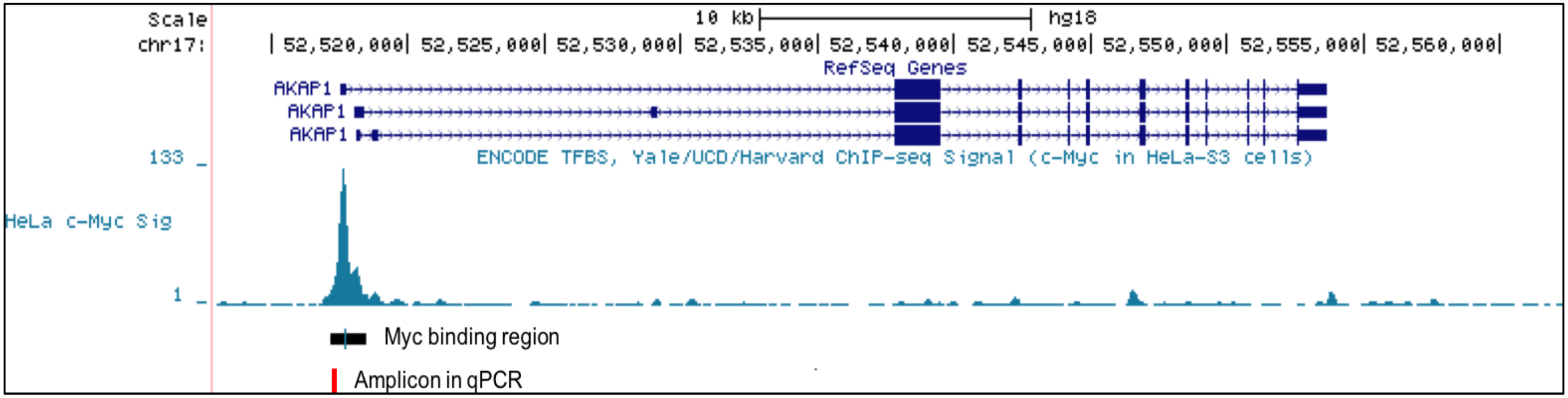

S1: A Screenshot of UCSC genome browser of AKAP1 gene is shown. The peak as well as the black bar represent the Myc binding on the promoter region and TSS of AKAP1 gene. Red bar shows the position of the amplicon used in qPCR for ChIP analysis.

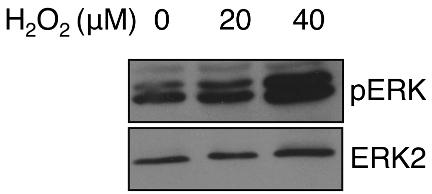

Supplementary Fig. S2. Lysates from HEK293 cells treated with hydrogen peroxide were immunoblotted for phospho-ERK1/2 and ERK2.

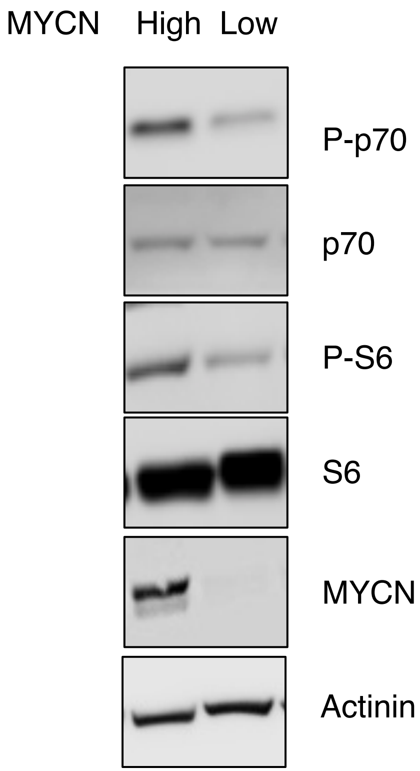

Supplementary Fig. S3. MYCN activates mTOR. Lysates from Tet21/N MYCN Tet-Off cells with Off (tetracyclin-treated cells) and On (untreated cells) MYCN were immunoblotted with the indicated antibodies.

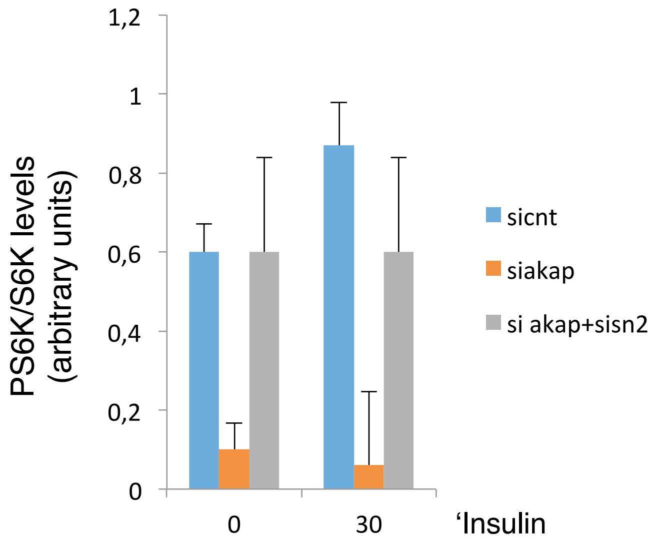

Supplementary Fig. S4. Quantitative analysis of the experiment shown in the Figure 4i. A mean value of three independent experiments is shown.

a

Characteristics	No, of cases(%)	No, of cases(%) AKAP1+
**All cases**	94	87
**Age, years**		
>60 y	61(64.8%)	57(65.5%)
<60 y	33(35,1%)	30(34.5%)
**Gender**		
male	66(70.2%)	61(70.1%)
female	28(29.8%)	26(29.9%)
**Disease stage**		
IA	17(18.0%)	16(18.4%)
IB	31(33.0%)	29(33.3%)
IIA	10(10.6%)	10(11.5%)
IIB	13(13.8%)	12(13.8%)
IIIA	11(11.7%)	9(10.3%)
IV	2(2.1%)	2(2.3%)
NA*	10(10.6%)	9(10.3%)
**Grade**		
I	4(4.2%)	3(3.4%)
II	48(51.0%)	42(48.3%)
III	16(17.0%)	16(18.4%)
NA*	26(27.7%)	26(29.9%)
**Subtype**		
ADCs	48(51.0%)	47(54.0%)
SCCs	29(30.8%)	25(28.7%)
OTHER	17(18.2%)	15(17.2%)

b

		pmTOR		Tot
		neg	pos	
AKAP1	neg	7	0	7
	pos	59	22	81
Tot		66	22	88

p=0.075

c

		cMyc		Tot
		neg	pos	
AKAP1	neg	7	0	7
	pos	77	10	87
Tot		84	10	94

p>0.05

Supplementary Fig. S5. Clinical and pathological features of NSCLC patients. (**a**) A total of 94 NSCLC (Non-Small Cell Lung Cancer) were analyzed. They consisted of 66 males, and 28 females. Mean age was 60 years (range 44-82). 48 tumors were adenocarcinoma (ADCs), and 29 tumors were squamous cell carcinoma (SCCs). The immunohistochemical analysis was conducted on a totale of 87 tumors. A score of 0-3 was given. It shows that AKAP1 was overexpressed in 57 out 87 tumors (65.5%). The clinical and pathological features of 87 NSCLC patients with AKAP1 expression are shown in **Table 2**. 45 out of 87 AKAP1-positive patients were stage I, 22/87 stage II, 9/87 stage III and 2/87 stage IV. 42 cases out of 87 were grade II, 16/87 grade III and 3/87 grade I. 47 cases out of 87 were adenocarcinomas, a strong statistical significance has been shown for AKAP1 expression in adenocarcinoma histotype (*P*=0.004). (**b-c**) Correlative analysis of the immunoreactivity for phospho-mTOR (b) and Myc (c) in AKAP1-positive and AKAP1-negative NSCLC tissue sections.
